# Supplementary figures and images for: COVID-19 related posttraumatic stress disorder in children and adolescents in Saudi Arabia
Source: PLoS One. 2021 Aug 4;16(8):e0255440. doi: 10.1371/journal.pone.0255440 (PMC8336789; doi:10.1371/journal.pone.0255440)

**S1 Fig. PASS output for calculation of the power of study.**

**
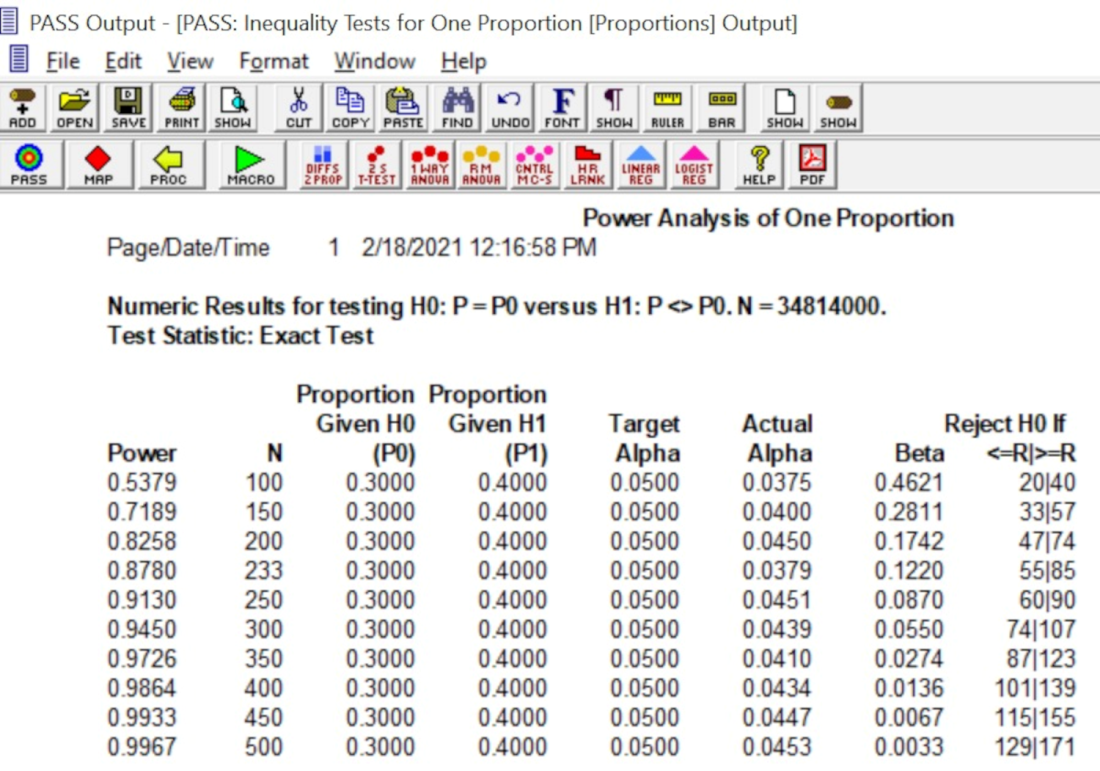
**

Supplement: S1 Fig — (DOCX) [file pone.0255440.s001.docx]
