# Supplementary material for: COVID-19 related posttraumatic stress disorder in children and adolescents in Saudi Arabia
Source: PLoS One. 2021 Aug 4;16(8):e0255440. doi: 10.1371/journal.pone.0255440 (PMC8336789; doi:10.1371/journal.pone.0255440)
Supplement: S1 Table — (DOCX) [file pone.0255440.s002.docx]

**S1 Table. Upsetting issues associated with or caused by COVID-19**

| **Upsetting issue** | | **n** | **%** |
| --- | --- | --- | --- |
|  | Being away from some family members (because of isolation or lockdown in other country) | 12 | 23.5 |
|  | Home stay/isolation from outside world for long period | 11 | 21.6 |
|  | Work/isolation of mother/father in health care center | 5 | 9.8 |
|  | Inability to visit grandfather and grandmother | 4 | 7.8 |
|  | Fear of contact with sick people | 4 | 7.8 |
|  | Being away from my parents for longtime | 3 | 5.9 |
|  | Difficulty tin moving between regions (eg. lockdown in Makkah) | 3 | 5.9 |
|  | Missing school/ meeting with friends | 2 | 3.9 |
|  | Closing of shops | 2 | 3.9 |
|  | Psychological effect of fear (anxiety, depression) | 2 | 3.9 |
|  | Delay of a surgery | 1 | 2.0 |
|  | Comorbidity (IBS) | 1 | 2.0 |
|  | Loss of job/financial insecurity | 1 | 2.0 |
