# Supplementary material for: COVID-19 related posttraumatic stress disorder in children and adolescents in Saudi Arabia
Source: PLoS One. 2021 Aug 4;16(8):e0255440. doi: 10.1371/journal.pone.0255440 (PMC8336789; doi:10.1371/journal.pone.0255440)
Supplement: S2 Table — (DOCX) [file pone.0255440.s003.docx]

**S2 Table. Frequency Distribution of University of California at Los Angeles Brief COVID-19 Screen for Child/Adolescent PTSD Questionnaire Variables**

|  | Never happened (None per month) | | Little or Happened twice per month | | Some or It happened once to twice per week | | Much or It happened two to three times per week | | Most or It happened almost every day | |
| --- | --- | --- | --- | --- | --- | --- | --- | --- | --- | --- |
|  | n | (%) | n | (%) | n | (%) | n | (%) | n | (%) |
| Q1. I try to stay away from people, places or things that remind me about what happened or what is still happening | 251 | 46.7 | 58 | 10.8 | 65 | 12.1 | 66 | 12.3 | 97 | 18.1 |
| Q2. I get upset easily, or get into arguments, or physical fights | 286 | 53.3 | 139 | 25.9 | 73 | 13.6 | 28 | 5.2 | 11 | 2.0 |
| Q3. I have trouble concentrating or paying attention | 288 | 53.6 | 145 | 27.0 | 77 | 14.3 | 18 | 3.4 | 9 | 1.7 |
| Q4. When something reminds me of what happened or is still happening, I get very upset, afraid or sad | 228 | 42.5 | 150 | 27.9 | 79 | 14.7 | 58 | 10.8 | 22 | 4.1 |
| Q5. I have trouble feeling happiness or love | 306 | 57.0 | 113 | 21.0 | 64 | 11.9 | 37 | 6.9 | 17 | 3.2 |
| Q6. I try not to think about or have feelings about what happened or is still happening | 226 | 42.1 | 131 | 24.4 | 92 | 17.1 | 55 | 10.2 | 33 | 6.1 |
| Q7. When something reminds me of what happened, I have strong feelings in my body like heart rate beats fast, my head aches or my stomach aches | 328 | 61.1 | 102 | 19.0 | 69 | 12.8 | 31 | 5.8 | 7 | 1.3 |
| Q8. I have thoughts like “I will never be able to trust other people” | 284 | 52.9 | 134 | 25.0 | 53 | 9.9 | 51 | 9.5 | 15 | 2.8 |
| Q9. I feel alone even when I am around other people | 322 | 60.0 | 106 | 19.7 | 51 | 9.5 | 36 | 6.7 | 22 | 4.1 |
| Q10. I have upsetting thoughts, pictures or sounds of what happened or is still happening come into my mind when I don’t want them to | 337 | 62.8 | 109 | 20.3 | 42 | 7.8 | 34 | 6.3 | 15 | 2.8 |
| Q11. I have trouble going to sleep, wake up often, or have trouble getting back to sleep | 284 | 52.9 | 128 | 23.8 | 54 | 10.1 | 44 | 8.2 | 27 | 5.0 |
|  |  |  |  |  |  |  |  |  |  |  |
