# Supplementary material for: COVID-19 related posttraumatic stress disorder in children and adolescents in Saudi Arabia
Source: PLoS One. 2021 Aug 4;16(8):e0255440. doi: 10.1371/journal.pone.0255440 (PMC8336789; doi:10.1371/journal.pone.0255440)
Supplement: S3 Table — (DOCX) [file pone.0255440.s004.docx]

**S3 Table. Frequency distribution of intrusion category B symptoms in 4 PTSD categories**

| Category B symptoms | | | Q4 symptom present or not | | Q7 symptom present or not | | Q10 symptom present or not | |
| --- | --- | --- | --- | --- | --- | --- | --- | --- |
|  |  |  | N | % | N | % | N | % |
| rating 0 no PTSD symptom |  | No | 83 | 100.0 | 83 | 100.0 | 83 | 100.0 |
| rating 1-10 minimal PTSD symptom |  | No | 233 | 98.3 | 235 | 99.2 | 236 | 99.6 |
|  |  | Yes | 4 | 1.7 | 2 | 0.8 | 1 | 0.4 |
|  |  | Total | 237 | 100.0 | 237 | 100.0 | 237 | 100.0 |
| rating 11-20 mild PTSD symptoms |  | No | 115 | 78.2 | 137 | 93.2 | 131 | 89.1 |
|  |  | Yes | 32 | 21.8 | 10 | 6.8 | 16 | 10.9 |
|  |  | Total | 147 | 100.0 | 147 | 100.0 | 147 | 100.0 |
| rating 21+ potential PTSD |  | No | 24 | 34.3 | 44 | 62.9 | 36 | 51.4 |
|  |  | Yes | 46 | 65.7 | 26 | 37.1 | 34 | 48.6 |
|  |  | Total | 70 | 100.0 | 70 | 100.0 | 70 | 100.0 |

*Symptom of Q4: Getting upset or sad when something remind me of what happened is the most frequent symptom in this category B symptoms and % increases with more total score of group ie. more with potential PTSD
