# Supplementary material for: COVID-19 related posttraumatic stress disorder in children and adolescents in Saudi Arabia
Source: PLoS One. 2021 Aug 4;16(8):e0255440. doi: 10.1371/journal.pone.0255440 (PMC8336789; doi:10.1371/journal.pone.0255440)
Supplement: S4 Table — (DOCX) [file pone.0255440.s005.docx]

**S4 Table. Frequency distribution of avoidance category C symptoms in 4 PTSD categories**

| Category C symptoms | | | Q1 symptom present or not | | Q6 symptom present or not | |
| --- | --- | --- | --- | --- | --- | --- |
|  |  |  | N | % | N | % |
| rating 0 no PTSD symptom |  | No | 83 | 100.0 | 83 | 100.0 |
| rating 1-10 minimal PTSD symptom |  | No | 186 | 78.5 | 225 | 94.9 |
|  |  | Yes | 51 | 21.5 | 12 | 5.1 |
|  |  | Total | 237 | 100.0 | 237 | 100.0 |
| rating 11-20 mild PTSD symptoms |  | No | 78 | 53.1 | 108 | 73.5 |
|  |  | Yes | 69 | 46.9 | 39 | 26.5 |
|  |  | Total | 147 | 100.0 | 147 | 100.0 |
| rating 21+ potential PTSD |  | No | 26 | 37.1 | 33 | 47.1 |
|  |  | Yes | 44 | 62.9 | 37 | 52.9 |
|  |  | Total | 70 | 100.0 | 70 | 100.0 |

*Symptom of Q1: I try to stay away from people, places or things that remind me about what happened or what is still happening is the most frequent symptom in this category C symptoms and % increases with more total score of group ie. more with potential PTSD
