# Supplementary material for: COVID-19 related posttraumatic stress disorder in children and adolescents in Saudi Arabia
Source: PLoS One. 2021 Aug 4;16(8):e0255440. doi: 10.1371/journal.pone.0255440 (PMC8336789; doi:10.1371/journal.pone.0255440)
Supplement: S5 Table — (DOCX) [file pone.0255440.s006.docx]

**S5 Table. Frequency distribution of negative conditions/mood category D symptoms in 4 PTSD categories**

| Category D symptoms | | | Q5 symptom present or not | | Q8 symptom present or not | | Q9 symptom present or not | |
| --- | --- | --- | --- | --- | --- | --- | --- | --- |
|  |  |  | N | % | N | % | N | % |
| rating 0 no PTSD symptom |  | No | 83 | 100.0 | 83 | 100.0 | 83 | 100.0 |
| rating 1-10 minimal PTSD symptom |  | No | 235 | 99.2 | 234 | 98.7 | 230 | 97.0 |
|  |  | Yes | 2 | 0.8 | 3 | 1.3 | 7 | 3.0 |
|  |  | Total | 237 | 100.0 | 237 | 100.0 | 237 | 100.0 |
| rating 11-20 mild PTSD symptoms |  | No | 137 | 93.2 | 124 | 84.4 | 132 | 89.8 |
|  |  | Yes | 10 | 6.8 | 23 | 15.6 | 15 | 10.2 |
|  |  | Total | 147 | 100.0 | 147 | 100.0 | 147 | 100.0 |
| rating 21+ potential PTSD |  | No | 28 | 40.0 | 31 | 44.3 | 34 | 48.6 |
|  |  | Yes | 42 | 60.0 | 39 | 55.7 | 36 | 51.4 |
|  |  | Total | 70 | 100.0 | 70 | 100.0 | 70 | 100.0 |

*Symptom of Q5: I have trouble feeling happiness or love, more with potential PTSD and % increases with more total score group ie. more with potential PTSD
