# Supplementary material for: COVID-19 related posttraumatic stress disorder in children and adolescents in Saudi Arabia
Source: PLoS One. 2021 Aug 4;16(8):e0255440. doi: 10.1371/journal.pone.0255440 (PMC8336789; doi:10.1371/journal.pone.0255440)
Supplement: S6 Table — (DOCX) [file pone.0255440.s007.docx]

**S6 Table. Frequency distribution of** **arousal/reactivity symptoms category E symptoms in 4 PTSD categories**

| Category E symptoms | | | Q2 symptom present or not | | Q3 symptom present or not | | Q11 symptom present or not | |
| --- | --- | --- | --- | --- | --- | --- | --- | --- |
|  |  |  | N | % | N | % | N | % |
| rating 0 no PTSD symptom |  | No | 83 | 100.0 | 83 | 100.0 | 83 | 100.0 |
| rating 1-10 minimal PTSD symptom |  | No | 223 | 94.1 | 235 | 99.2 | 229 | 96.6 |
|  |  | Yes | 14 | 5.9 | 2 | 0.8 | 8 | 3.4 |
|  |  | Total | 237 | 100.0 | 237 | 100.0 | 237 | 100.0 |
| rating 11-20 mild PTSD symptoms |  | No | 93 | 63.3 | 143 | 97.3 | 118 | 80.3 |
|  |  | Yes | 54 | 36.7 | 4 | 2.7 | 29 | 19.7 |
|  |  | Total | 147 | 100.0 | 147 | 100.0 | 147 | 100.0 |
| rating 21+ potential PTSD |  | No | 27 | 38.6 | 49 | 70.0 | 37 | 52.9 |
|  |  | Yes | 43 | 61.4 | 21 | 30.0 | 33 | 47.1 |
|  |  | Total | 70 | 100.0 | 70 | 100.0 | 70 | 100.0 |

Symptom of Q 2 is considered present if it happened once to twice per week or more (frequency score ≥ 2)

All other symptoms of PTSD are considered present if they happened 2-3 per week or almost every day (frequency score 3 or 4)

*Symptom of Q 2: I get upset easily, or get into arguments, or physical fights, is the most frequent symptom in this category E and % increases with more total score of group ie. more with potential PTSD
