# Supplementary material for: COVID-19 related posttraumatic stress disorder in children and adolescents in Saudi Arabia
Source: PLoS One. 2021 Aug 4;16(8):e0255440. doi: 10.1371/journal.pone.0255440 (PMC8336789; doi:10.1371/journal.pone.0255440)
Supplement: S7 Table — (DOCX) [file pone.0255440.s008.docx]

**S7 Table. Univariate analysis of risk factors between categories of PTSD**

| Variable | No PTSD symptom (n=83)  N % | Minimal PTSD symptom  (n=237)  N % | Mild PTSD symptoms  (n=147)  N % | Potential PTSD  (n=70)  N % | X^2^ P |
| --- | --- | --- | --- | --- | --- |
| Nationality  Saudi  Non Saudi | 78 94.0  5 6.0 | 223 94.1  14 5.9 | 135 91.8  12 8.2 | 58 82.9  12 17.1 | 9.7 0.02 |
| Region  Central region  Western region  Eastern region  Northern region  Southern region | 7 8.4  70 84.3  2 2.4  4 4.8  0 0 | 20 8.4  185 78.1  13 5.5  10 4.2  9 3.8 | 8 5.4  124 84.4  3 2.0  8 5.4  4 2.7 | 13 18.6  54 77.1  1 1.4  1 1.4  1 1.4 | 20.4 0.06 |
| Age  7-12  13-18 | 47 56.6  36 43.4 | 115 48.5  122 51.5 | 72 49.0  75 51.0 | 42 60.0  28 40.0 | 4.1 0.25 |
| Gender  Boy  Girl | 46 55.4  37 44.6 | 117 49.4  120 50.6 | 65 44.2  82 55.8 | 34 48.6  36 51.4 | 2.7 0.44 |
| Number of children/family  1  2  3  4  5  >5 | 15 18.1  16 19.3  15 18.1  17 20.5  13 15.7  7 8.4 | 32 13.5  52 21.9  48 20.3  50 21.1  34 14.3  21 8.9 | 20 13.6  44 29.9  29 19.7  18 12.2  12 8.2  24 16.3 | 9 12.9  21 30.0  11 15.7  18 25.7  5 7.1  6 8.6 | 22.6 0.09 |
| School level  Primary  Intermediate  Secondary | 41 49.4  16 19.3  26 31.3 | 109 46.0  58 24.5  70 29.5 | 68 46.3  35 23.8  44 29.9 | 41 58.6  11 15.7  18 25.7 | 4.7 0.58 |
| Illness of child/close relative because of COVID-19  No  Yes | 81 97.6  2 2.4 | 226 95.4  11 4.6 | 144 98.0  3 2.0 | 66 94.3  4 5.7 | 2.9 0.41 |
| Quarantine of child/close relative because of COVID-19  No  Yes | 81 97.6  2 2.4 | 224 94.5  13 5.5 | 142 96.6  5 3.4 | 65 92.9  5 7.1 | 2.8 0.42 |
| Positive SARS-CoV-2 of child/close relative  No  Yes | 80 96.4  3 3.6 | 222 93.7  15 6.3 | 138 93.9  9 6.1 | 61 87.1  9 12.9 | 5.6 0.13 |
| Work of close relative around people who might have COVID-19  No  Yes | 72 86.7  11 13.3 | 165 69.6  72 30.4 | 97 66.0  50 34.0 | 43 61.4  27 38.6 | 14.7 0.002 |
| Moving of a family member away from home because of COVID-19  No  Yes | 81 97.6  2 2.4 | 224 94.5  13 5.5 | 140 95.2  7 4.8 | 64 91.4  6 8.6 | 3.0 0.39 |
| Death of any close relative/friend because of COVID-19  No  Yes | 81 97.6  2 2.4 | 229 96.6  8 3.4 | 141 95.9  6 4.1 | 65 92.9  5 7.1 | 2.6 0.45 |
| Anything else happened to you or your family because of SARS-CoV-2  No  Yes | 80 96.4  3 3.6 | 218 92.0  19 8.0 | 123 83.7  24 16.3 | 60 85.7  10 14.3 | 12.2 0.007 |
