# Supplementary material for: COVID-19 related posttraumatic stress disorder in children and adolescents in Saudi Arabia
Source: PLoS One. 2021 Aug 4;16(8):e0255440. doi: 10.1371/journal.pone.0255440 (PMC8336789; doi:10.1371/journal.pone.0255440)
Supplement: S8 Table — (DOCX) [file pone.0255440.s009.docx]

**S8 Table. Multinomial regression for all studied variables or risk factors associated with potential PTSD compared to the other 3 combined groups (Group without PTSD, Group with minimal and Group with mild PTSD)**

NB: Only Saudi nationality persisted as significant factor with significantly more Saudi children with potential PTSD than non-Saudi children with potential PTSD (as certainly number of Saudi participants were significantly more non Saudi).

| PTSD2GP^a^ | | B | Wald | Sig. | Exp(B) | 95% Confidence Interval for Exp(B) | |
| --- | --- | --- | --- | --- | --- | --- | --- |
|  |  |  |  |  |  | Lower Bound | Upper Bound |
| Potential | Intercept | -0.30 | 0.05 | 0.83 |  |  |  |
|  | [Nationality =1] | -0.94 | 5.42 | 0.02 | 0.39 | 0.17 | 0.86 |
|  | [Nationality =2] |  | . | . | . | . | . |
|  | [Region=1.00] | 1.51 | 1.71 | 0.19 | 4.53 | 0.47 | 43.50 |
|  | [Region=2.00] | 0.67 | 0.37 | 0.54 | 1.96 | 0.23 | 16.99 |
|  | [Region=3.00] | -0.31 | 0.04 | 0.84 | 0.73 | 0.04 | 14.17 |
|  | [Region=4.00] | -0.60 | 0.16 | 0.69 | 0.55 | 0.03 | 10.58 |
|  | [Region=5.00] |  | . | . | . | . | . |
|  | [How many children do you have=1] | -0.08 | 0.02 | 0.89 | 0.92 | 0.28 | 3.01 |
|  | [How many children do you have=2] | 0.31 | 0.33 | 0.56 | 1.37 | 0.47 | 3.98 |
|  | [How many children do you have=3] | -0.24- | 0.18 | 0.67 | 0.80 | 0.26 | 2.41 |
|  | [How many children do you have=4] | 0.60 | 1.24 | 0.27 | 1.82 | 0.63 | 5.22 |
|  | [How many children do you have=5] | -0.28 | 0.18 | 0.67 | 0.75 | 0.20 | 2.80 |
|  | [How many children do you have=6] |  | . | . | . | . | . |
|  | [Agegroup=1] | -0.13 | 0.03 | 0.87 | 0.88 | 0.19 | 4.10 |
|  | [Agegroup=2] |  | . | . | . | . | . |
|  | [Gender of Child =1] | 0.10 | 0.05 | 0.82 | 1.06 | 0.62 | 1.82 |
|  | [Gender of Child =2] | 0^b^ | . | . | . | . | . |
|  | [studylevel=1.00] | 0.44 | 0.27 | 0.60 | 1.56 | 0.30 | 8.00 |
|  | [studylevel=2.00] | -0.32 | 0.50 | 0.48 | 0.73 | 0.30 | 1.76 |
|  | [studylevel=3.00] |  | . | . | . | . | . |
|  | [Have you (the son / daughter) or someone close to you gotten sick or been in hospital because of this virus?=0] | 0.41 | 0.21 | 0.65 | 1.50 | 0.25 | 8.90 |
|  | [Have you (the son / daughter) or someone close to you gotten sick or been in hospital because of this virus?=1] |  | . | . | . | . | . |
|  | [Have you (the son / daughter) or someone close to you been quarantined because of symptoms of this virus?=0] | -0.19 | 0.06 | 0.81 | 0.83 | 0.18 | 3.80 |
|  | [Have you (the son / daughter) or someone close to you been quarantined because of symptoms of this virus?=1] |  | . | . | . | . | . |
|  | [Have you (the son / daughter) or someone close to you been told a positive test for this virus?=0] | -0.89 | 2.45 | 0.12 | 0.41 | 0.135 | 1.25 |
|  | [Have you (the son / daughter) or someone close to you been told a positive test for this virus?=1] |  | . | . | . | . | . |
|  | [Does someone close to you work around people who might have this virus?=0] | -0.18 | 0.38 | 0.55 | 0.84 | 0.48 | 1.47 |
|  | [Does someone close to you work around people who might have this virus?=1] |  | . | . | . | . | . |
|  | [Have you or a family member had to move away from home because of this virus?=0] | -0.53 | 0.95 | 0.33 | 0.59 | 0.20 | 1.71 |
|  | [Have you or a family member had to move away from home because of this virus?=1] |  | . | . | . | . | . |
|  | [Has anyone close to you died because of this virus?=0] | -0.34 | 0.36 | 0.55 | 0.71 | 0.23 | 2.20 |
|  | [Has anyone close to you died because of this virus?=1] |  | . | . | . | . | . |
|  | [Has anything else happened to you or your family because of this virus and that has been upsetting?=0] | -0.20 | 0.20 | 0.66 | 0.84 | 0.37 | 1.87 |
|  | [Has anything else happened to you or your family because of this virus and that has been upsetting?=1] |  | . | . | . | . | . |
