# Supplementary material for: COVID-19 related posttraumatic stress disorder in children and adolescents in Saudi Arabia
Source: PLoS One. 2021 Aug 4;16(8):e0255440. doi: 10.1371/journal.pone.0255440 (PMC8336789; doi:10.1371/journal.pone.0255440)
Supplement: Attachment — Submitted filename: Reply to Reviewer Comments.docx [file pone.0255440.s011.docx]

**Dear Editor in Chief and Academic Editor of PLOS ONE Journal,**

Thank you for the second revision of this original article (PONE-D-21-07795: COVID-19 related posttraumatic stress disorder in children and adolescents in Saudi Arabia) and giving us the chance to respond and reply to all concerns of the reviewer to provide the most accurate presentation of the results of this original research and significantly improving the written manuscript.

We have responded to all comments and each point raised by the reviewer and all required necessary changes were added to the manuscript appropriately at the relevant sites highlighted in red color and within the requested time frame for revision.

Both a marked-up copy of the manuscript with highlighted changes (Revised Manuscript with Track Changes) and an unmarked version of the revised paper without tracked changes labelled (Manuscript) were uploaded as 2 separate files.

**Reply to the Comments of the Reviewer:**

1. Regarding the question about linear regression model (as opposed to the multinomial model), the reviewer suggested to adapt a linear regression model (preferably with fixed AND random effect using PTSD symptom count as dependent variable because this has much more power to detect small effects and allows to adequately correcting for hierarchical biases as introduced by rater and or language.

Response/Reply:

We have pointed out that this was related to the type of the studied variables included in the analysis or comparisons such as nationality (whether Saudi citizen or Non-Saudi resident), region (whether participants were from central, eastern, western, northern or southern region), age group (7-12 year old school children versus 13-18 year older adolescents), gender (either boy or girl) and study level (primary versus intermediate versus secondary school level). All the previously mentioned variables are represented in a qualitative way (whether present (Yes) or absent (No) and not in quantitative way in the form of (mean or median, standard deviation and range).

Additionally and unfortunately the dependent or outcome variable (PTSD symptoms count or scale score) in this study is not normally distributed and the problem is that the results of the parametric tests including t-test generally used in linear regression for analysis, will affect the significance and reliability of the regression equation and its parameters.

Moreover, normality violation and use of non-normally distributed dependent variable in linear regression, will affect the estimates of the standard error and the confidence interval, and hence the significance of the risk factors (independent variables or predictors).

Finally, linear regression techniques still can be used even if normality is violated using a dependent variable that is not distributed normally and linear regression remains a statistically sound technique in studies of large sample sizes which provides appropriate sample sizes (i.e., >3000) and this extremely large sample size was not available in this study (Li et al, 2012).

Li X, Wong W, Lamoureux EL, Wong TY. Are linear regression techniques appropriate for analysis when the dependent (outcome) variable is not normally distributed? Invest Ophthalmol Vis Sci. 2012 May 1; 53(6):3082-3. doi: 10.1167/iovs.12-9967.

2. Regarding using a questionnaire in two different languages as translation and cultural adaption of a questionnaire can bias a result. The authors should mention this in the limitation section or detail their efforts in the methods and supplements.

Response/Reply:

In the first revision, we confirmed that necessary measures were undertaken to avoid potential bias that may arise as a result of Arabic translation and cultural difference as it was mentioned in the materials and methods section of the originally submitted manuscript under the subheading; Questionnaire implementation and distribution, lines 143-145, that: The UCLA-BCSCA is originally available in English and it was translated into Arabic, checked by two bilingual experts and used in a pilot study for Arabic speaking participants to detect if any amendments are required. However, the potential limitation of using a questionnaire in two different languages as translation and cultural adaption of a questionnaire can bias a result was also clearly mentioned in the limitations of the study.

3. Regarding the potential bias between self and parent rating to ensure that language and/or rater do not influence the finding.

Response/Reply:

The potential bias between self and parent rating was added to study limitations.

4. Regarding data sharing: The reviewer was concerned about making all data underlying the findings in the manuscript fully available.

Response/Reply: The data was provided as part of the manuscript and in the supplementary materials or supporting information. Additionally, it was clearly mentioned in the first revision that the original raw data SPPS file was made available without restriction, and deposited to figshare public data repository. DOI: [10.6084/m9.figshare.14565531](https://doi.org/10.6084/m9.figshare.14565531) where it can be downloaded for replication purpose.

5. Very minor comment: During the revision process some minor typos have slipped in (e.g. line 101)

Response/reply: Line 101 was rechecked.
